# A Soft Sensor for Bioprocess Control Based on Sequential Filtering of Metabolic Heat Signals

**DOI:** 10.3390/s141017864

**Published:** 2014-09-26

**Authors:** Dan Paulsson, Robert Gustavsson, Carl-Fredrik Mandenius

**Affiliations:** Division of Biotechnology/IFM, Linköping University, Linköping 581 83, Sweden; E-Mails: 10809@hotmail.se (D.P.); robgu@ifm.liu.se (R.G.)

**Keywords:** bioprocess control, bio-calorimetry, software sensors, soft sensor implementation, bioprocess user interface

## Abstract

Soft sensors are the combination of robust on-line sensor signals with mathematical models for deriving additional process information. Here, we apply this principle to a microbial recombinant protein production process in a bioreactor by exploiting bio-calorimetric methodology. Temperature sensor signals from the cooling system of the bioreactor were used for estimating the metabolic heat of the microbial culture and from that the specific growth rate and active biomass concentration were derived. By applying sequential digital signal filtering, the soft sensor was made more robust for industrial practice with cultures generating low metabolic heat in environments with high noise level. The estimated specific growth rate signal obtained from the three stage sequential filter allowed controlled feeding of substrate during the fed-batch phase of the production process. The biomass and growth rate estimates from the soft sensor were also compared with an alternative sensor probe and a capacitance on-line sensor, for the same variables. The comparison showed similar or better sensitivity and lower variability for the metabolic heat soft sensor suggesting that using permanent temperature sensors of a bioreactor is a realistic and inexpensive alternative for monitoring and control. However, both alternatives are easy to implement in a soft sensor, alone or in parallel.

## 1. Introduction

**Figure 1.**
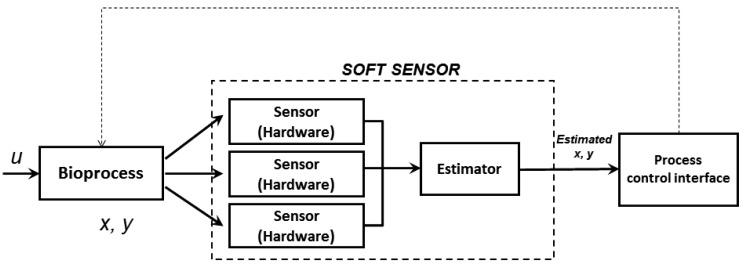
Basic principle of a soft sensor.

Soft sensors are frequently used for on-line estimations based on analysis of measurement signals from hardware sensors with software implemented mathematical models (Figure 1) [1,2]. Typically, the modeling is carried out with first principle models, regression models or artificial neural networks [3–6]. Soft sensors have successfully been applied in monitoring and control of large scale industrial processes [3]. In the biotechnology industry, however, applications are so far limited to demonstrations mostly at research scale [7–11]. An important aspect of soft sensors for industrial application is the availability of robust hardware sensors with appropriate signal quality for the soft models. Another important aspect is that the models should be satisfactorily validated for the sensor signals when implemented in a process monitoring control system [1].

Estimation of growth from the metabolic heat produced in a bioreactor culture has been applied for a variety of microbial and animal cells [12–19]. In most cases, this has been realized by recording the cooling effect that is needed for balancing the metabolic heat produced by the culture. From this accurate growth data are possible to derive in models based on energy balances [10,11]. Of particular interest is the derivation of specific growth rate and from that, the concentration of actively growing biomass in the bioreactor [12–17]. This makes soft sensors based on a metabolic heat model interesting alternatives to other soft sensor combinations, e.g., using CO_2_/O_2_ sensor signals in a respiration model [20,21]. However, the measurability and accuracy of the estimations are strongly related to the amount of heat produced by the culture, thus favoring high density cultivations, fast-growing organisms and large volume bioreactor systems. This requires signal processing and digital filtering of the obtained data. One of the interesting applications is to use the heat derived signals for process control purposes, such as feeding of media [14,15]. Other useful applications are monitoring of the progression of growth and protein production [15–17].

The Process Analytical Technology (PAT) initiative encourages exploitation of scientific understanding and control of the bioprocess with help of on-line sensors [22]. Key PAT objectives are to reduce variability of quality attributes and by that control of production at identified optimal conditions and to increase the degree of automation in manufacturing operations. Soft sensors in particular may effectively contribute to these objectives by controlling growth of biomass. The utilization of metabolic heat for that purpose is therefore a challenging technical possibility [18].

Several attempts have previously been carried out to improve the performance of metabolic heat sensors by digitally filtering the estimated biomass and specific growth rate derived from energy balance equations. Marison *et al.* [23] suggested the use of various digital filtering methods to improve the resolution and reduce the noise of the bio-calorimetric signals by frequent data sampling combined with mathematical smoothing procedures, such as Kalman filtering or floating point averaging.

Dabros *et al.* [24] presented recently a moving average filter for attaining estimates of higher stability and as a result, facilitating the use of the estimated μ for feedback control of a bioreactor yeast culture. The same research group extended the same type of average filter to other microorganisms [19]. These attempts have shown that it is possible to use the average filters with relatively long filter periods if the microorganisms have moderate growth rates [18], but the results also indicate the need for further improvement of the filtering procedure when using cultures with higher growth rates or with signals containing noise from the system.

The moving average filtering methods, such as low-pass filters, Savitzky-Golay filters and extended Kalman filters [25–27], applied in these previous studies are in this article further improved by applying a sequential filtering method. We suggest that this method is of great benefit, in particular when conditions in the bioreactor are unfavorable, either due to small metabolic heat effects, to bioreactor constructions generating substantial noise effects or when a bioreactor culture grows at low rates.

The monitoring design in this article handles the sensor system with its accompanying models as a soft sensor in which the sequential filter efficiently reduces noise from the measurements. The soft sensor is implemented on a commercial computer control system in parallel to other monitoring and control functions in a fashion feasible for PAT applications. The soft sensor was applied for controlling a fed-batch *E. coli* cultivation producing a recombinant protein, green fluorescence protein (GFP), using a conventional proportional and integral controller. The soft sensor estimates were compared with an alternative on-line hardware sensor for cell viability concentration, a capacitance probe for cell viability.

## 2. Experimental Section

### 2.1. Cultivation

The *Escherichia coli* strain HMS 174(DE3) (Novagen, Madison, WI, USA) transformed with plasmid pET30a (Novagen) containing GFP-mut3.1 (Clontech, Mountain View, CA, US), under control of the *T7/lac* promoter and a 25 bp *lac* operator sequence was used. The transformed strain was obtained from the Department of Biotechnology, University of Natural Resources and Life Science, Vienna.

A semi-synthetic medium was used for the pre-culture while a modified medium was used in the fed-batch cultivation. All reagents and chemicals were purchased from Merck, if not otherwise stated. Media components were added in relation to the working volume of the bioreactor. The pre-culture medium was composed of 3.0 g·L^−1^ KH_2_PO_4_, 4.5 g·L^−1^ K_2_HPO_4_·3H_2_O, 2.5 g·L^−1^ C_6_H_5_Na_3_O_7_·2H_2_O, 1.0 g·L^−1^ MgSO_4_·7H_2_O, 4.5 g·L^−1^ (NH_4_)_2_SO_4_, 3.7 g·L^−1^ NH_4_Cl, 1.5 g·L^−1^ yeast extract, 6.0 g·L^−1^ glucose and 0.5 mL·L^−1^ of a trace element solution. The batch medium was composed of 5.0 g·L^−1^ glucose, 6.67 g·L^−1^ K_2_HPO_4_, 0.25 g·L^−1^ KH_2_PO_4_, 1.2 g·L^−1^ NaCl, 1.1 g·L^−1^ K_2_SO_4_, 0.5 g·L^−1^ yeast extract, 10 g·L^−1^ (NH_4_)_2_SO_4_, 0.15 g·L^−1^ MgSO_4_·7H_2_O, 0.013 g·L^−1^ CaCl_2_·2H_2_O and 0.125 mL·L^−1^ of the trace element solution, and the feed medium was composed of 100 g·L^−1^ glucose, 27.5 g·L^−1^ (NH_4_)_2_SO_4_, 1.5 g·L^−1^ MgSO_4_·7H_2_O, 0.026 g·L^−1^ CaCl_2_·2H_2_O and 2.50 mL·L^−1^ of the trace element solution. The trace element solution contained 40.0 g·L^−1^ FeSO_4_·7H_2_O, 10.0 g·L^−1^ MnSO_4_·H_2_O, 10.0 g·L^−1^ AlCl_3_·6H_2_O, 4.0 g·L^−1^ CoCl_2_, 2.0 g·L^−1^ ZnSO_4_·7H_2_O, 2.0 g·L^−1^ Na_2_MoO_2_·2H_2_O, 1.0 g·L^−1^ CuCl_2_·2H_2_O and 0.50 g·L^−1^ H_3_BO_3_ dissolved in 5 N HCl.

All cultivations were carried out in a 10 L *in situ* sterilized bioreactor (Model LMS 2002, Belach Bioteknik AB, Stockholm, Sweden) equipped with standard instrumentation. The control software (BioPhantom, version 2000; Belach Bioteknik AB, Stockholm, Sweden) was implemented with the soft sensor algorithms described below. A pre-culture was prepared from one cell bank vial in a shake flask with 200 mL medium and grown overnight to an OD value of 8–12. This culture was used to inoculate the bioreactor at an initial batch medium volume of 4 L. During the subsequent fed-batch 2 L of feeding medium was added. The pH was controlled at 7.0 ± 0.1 by addition of 1 M sulfuric acid or 20% ammonia. The aeration rate was 1 vvm and the temperature was 37 °C. Dissolved oxygen (DO) was controlled to 30% by adjusting the stirrer speed (300–1200 rpm). Foaming was controlled by the addition of a 50% anti-foam solution (Glanapon, Busetti & Co GmbH, Vienna, Austria).

Exponential feeding of the fed-batch was started 30 min after glucose depletion in the batch medium. This was a well-defined starting point which was easy to reproduce. The feeding rate of the feed pump (P4 U1-MXV, Alitea, Stockhom, Sweden) was controlled by the soft sensor (see below).

GFP expression was induced by addition of 0.03 g·L^−1^ isopropyl β-d-1-thiogalactopyranoside (IPTG) (Sigma) when the OD_600_ had reached a value of 40 absorbance units. This corresponded to approximately one culture generation and occurred 4 h after starting of feeding.

### 2.2. Instrumentation

**Figure 2.**
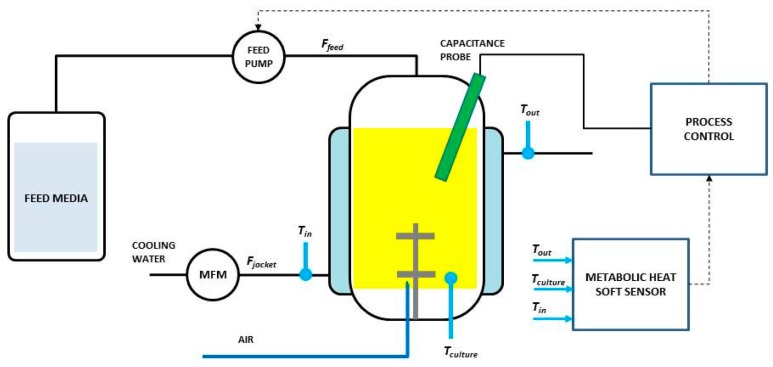
Experimental set-up of the soft sensor.

The reactor was equipped with standard DO and pH electrodes as well as probes for temperature, headspace pressure, and volume level (Figure 2). Temperature sensors (Belach Bioteknik AB, Stockholm, Sweden) were placed at influent and effluent pipes of the bioreactor jacket and inside at the bottom of the bioreactor vessel. Signals were acquired every second. Flow rate of cooling water through the jacket was measured and controlled by the BioPhantom software.

Cell viability was measured using a dielectric probe at a frequency of 1000 kHz (Standard Futura, Aber Instruments Ltd., Aberystwyth, UK). The 12 mm probe was placed *in situ* and top-mounted as seen in Figure 2. The capacitance signal from the probe was analyzed in the Aber Instruments' SCADA software on a separate PC which delivered the signal to the BioPhantom software.

### 2.3. Analyses

Samples for measurement of optical density at OD_600_ (using Ultraspec 1000, Pharmacia Biotech, UK) and the biomass dry weight were taken from the bioreactor intermittently through a steamed pipe. Diluted un-centrifuged GFP samples were measured in a fluorimeter (Fluostar Galaxy, BMG Labtechnologies GmbH, Offenburg, Germany) at excitation/emission wavelengths of 470/515 nm in 1-mL samples. As reference was used un-induced culture media. The fluorescence units of the fluorimeter were calibrated *vs.* a GFP standard solution (recombinant GFP; Millipore AB, Solna, Sweden).

## 3. Modeling and Sequential Filtering

A previously described calorimetric model for bioreactor processes by Voisard *et al*. [14], later adopted by several other researchers [15–19], was used in this study. The model assumes constant and controlled temperature around the bioreactor where a simplified heat flow balance is defined according to:

(1) $$q_{\text{metabol}}=q_{\text{jacket}}-q_{\text{stir}}+q_{\text{gas}}+q_{\text{feed}}+q_{\text{env}}$$

where *q_metabol_* is the metabolic heat flow (J·s^−1^) from the culture, *q_jacket_* the removal of heat by the flowing cooling water, and the terms *q_stir_* the heat from stirring, *q_gas_* the heat removal by the air flow through the reactor, *q_feed_* the cooling effect by addition of feed, and *q_env_* the loss of heat to the environment. Effects due to addition of ammonia for pH control and CO_2_ stripping through the gassing of the reactor are neglected, as previously shown [14]. Heat flows due to the gassing *per se* and to the loss to the environment are both considered constant in the cultivations and included in heat flow for stirring. As a result, (Equation (1)) is simplified to:

(2) $$q_{\text{metabol}}=q_{\text{jacket}}-q_{\text{stir}}+q_{\text{feed}}$$

The heat flow to the jacket *q_jacket_* is determined in the experiments by the temperature sensors that measure temperature of the influent (*T_in_*) and effluent (*T_out_*) cooling water to the bioreactor jacket.

(3) $$q_{\text{jacket}}=F_{\text{jacket}}\times c_{p}\times (T_{\text{out}}-T_{in})$$

where *F_jacket_* is the jacket water flow rate (L·s^−1^) and *c_p_* the heat capacity of water (4184 J·°C^−1^·L^−1^).

**Figure 3.**
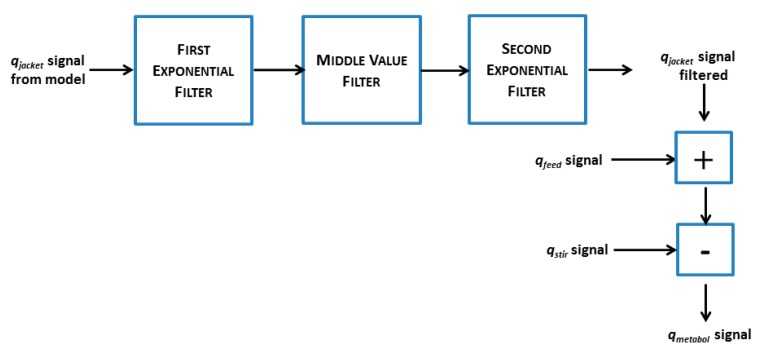
Sequential filtering steps of signal from the calorimetric model before used for monitoring and control of the fed-batch process.

Due to short term heat flow variations in the cooling systems of small-scale bioreactors we have extended this model with a digital filter based on a sequence of a first exponential filter, a middle-value filter and a second exponential filter (Figure 3). In the first exponential filter the *q_jacket_* value is acquired from (Equation (3)) and filtered according to:

(4) $$q_{\text{filt}er1,n}=q_{\text{filt}er1,n-1}\times a+(1-a)\times q_{\text{jacket},n}$$

where *q_filter_*_1_ is the heat signal after the first exponential filtering and *a* is the exponential filter constant according to:

(5) $$a=\frac{T_{\text{filt}}}{\left(T_{\text{filt}}+T_{\text{scan}}\right)}$$

where *T_filt_* is the filter time in seconds and *T_scan_* the scan time with a time interval between sample points of 0.25 s.

The subsequent middle-value filter adds the value of the peak and trough of the last period time of 7 min or 1680 sample points of *q_filter_*_1_ and divides by two according to:

(6) $${\text{q}}_{\text{filter}2,\text{n}}=\left\{\text{max}\left(q_{\text{filt}er1}\left[n-1680+1:n\right]\right)+\text{min}\left(q_{\text{filt}er1}\left[n-1680+1:n\right]\right)\right\}/2$$

The subsequent second exponential filter used Equations (5) and (6) as in the first exponential filter.

The heat flow to the added feed media due to the temperature difference between the feed tank and the reactor culture (Δ*T_feed_*) was considered constant and approximated to:

(7) $$q_{\text{feed}}=\Delta T_{\text{feed}}\times C_{p\text{feed}}\times F_{\text{feed}}/3600$$

where *C_p,feed_* is a heat capacity of the feed media (3.8845 J·g^−1^·°C^−1^) and *F_feed_* the feed rate (g·L^−1^).

The specific growth rate μ was estimated from the *q_metabol_*. We used the same assumption as previously described by Biener *et al*. [16,17] resulting in the simplified relation:

(8) $$\mu_{\text{metabol}}=\frac{1}{q_{\text{metabol}}}\times \frac{dq_{\text{metabol}}}{dt}$$

Biomass concentration is then derived from (Equation (8)) according to:

(9) $$X_{\text{metabol},n}=\left(X_{\text{metabol},n-1}\times V_{n-1}\right)\times e^{\mu_{\text{metabol},n-1}\times (t_{n}-t_{n-1})}/V_{n}$$

where *V* is the volume in the bioreactor (L).

In the control experiments the feed rate was controlled from the estimated specific growth rate using a conventional PI controller supplied with an adaptive factor possible to adjust during the cultivation:

(10) $$F_{\text{feed}}=F_{\text{start}}+K_{P}\left(e+\frac{1}{T_{I}}\int ed\tau \right)$$

where the error is:

(11) $$e=\mu_{\text{setpoint}}-\mu_{\text{metabol}}$$

and where *K_P_* and *T_I_* are controller parameters.

## 4. Implementation of the Soft Sensor Model in Process Control System

**Figure 4.**
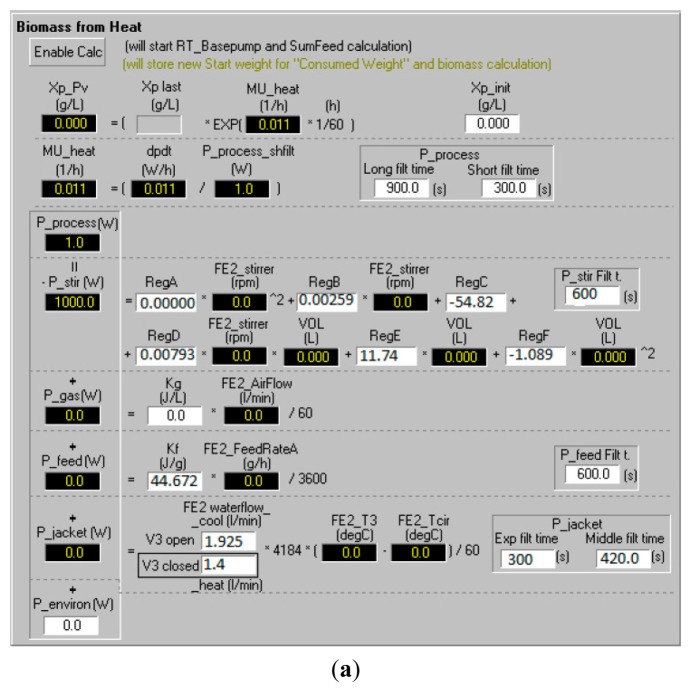

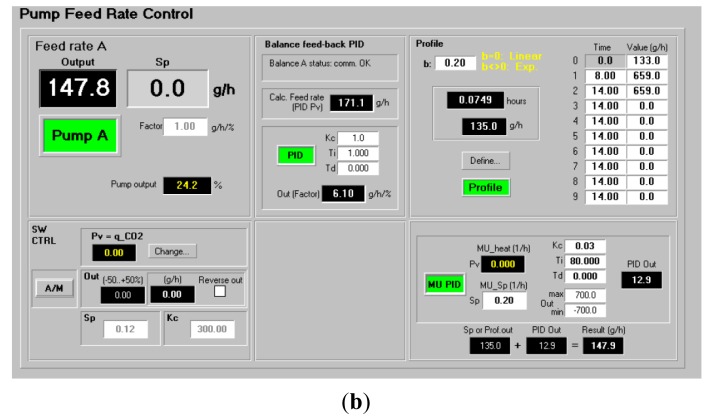
(**a**) The user interface of the soft sensor model (*P* = *q_heat_* W = J·s^−1^); (**b**) A sub-interface of the soft sensor model for setting the feed profile and parameters of the PID controller used for control *μ_metabol_* of the bioreactor cultivation.

The model equations described above were implemented on the bioreactor control system (BioPhantom, Belach Bioteknik AB, Stockholm, Sweden). An interactive interface was built where the operator could adjust the parameters of the calorimetric model, the control algorithm and other sensor parameters in the soft sensor (Figure 4).

The interface and its sub-windows provided the user with on-line estimations of specific growth rate and biomass concentration from the model and allowed easy exchange of pre-determined model parameters (see example of interface). Process control was carried out by basic PID control from fixed or dynamic set-points (Figure 4b). The interface also allowed combinations with other on-line signals from the bioreactor.

The *q_stir_* was fitted to stirrer speed from calibrations and a second order quadratic polynomial. This polynomial was included in the soft sensor configuration where regression constants determined in separate calibration runs at different of stirrer speeds and reactor volumes were set (see Figure 4a).

## 5. Results and Discussion

The purpose of the study was to apply the principle of monitoring and control from robust signals derived from standard bioreactor sensors and as a result, providing an example of a soft sensor configuration feasible for PAT application.

The sections below present results from the study highlighting the sequential filtering method, comparing estimates from metabolic heat with a sensor for direct monitoring of viable biomass and demonstrate the outcome of feedback control from the filtered metabolic heat estimates.

### 5.1. Signal Improvement by Sequential Filtering

The computed specific growth estimates from raw signal data showed considerable instability and noise. This was mainly ascribed to the design of the cooling system of the bioreactor, exacerbated by the low heat production of the low cell-density cultivation. Consequently, it was not a reflection of the actual metabolic activity of the cultivation which could be expected to shows a monotonically increasing metabolic activity during the feed phase. Furthermore, this signal behavior would cause severe instability if the soft sensor would be applied in a specific growth rate controller, as the specific growth rate computation includes the derivative of the *q_jacket_* signal (see Equation (5)). Especially the fact that within each periodic variation, oscillating variations in the heat signal led to negative *μ_heat_* that made the controller to act by increasing the feed rate. This was followed by a periodic increase in the heat signal and resulted in a large positive *μ_heat_* change that elicited a sudden controller action with a decrease in the feed rate that limited the control possibility. This periodic behavior of the heat signal called for a specially designed filtering solution since a common smoothing exponential filter would not be able to remove the long periodic variations.

Thus, the main goal of the sequential filtering of the metabolic heat signal applied here was to capture the increasing production of metabolic heat from the growing culture in the fed-batch bioreactor. The monitored heat profile should accurately show the characteristic shape of the exponential growth during the fed-batch operation based on the above described calorimetric model [14].

As a result, a heat profile should result that is mainly exponentially increasing as long as the feed rate is increasing. Importantly, the effect of major disturbances and other incidents that occur during the feed phase must be recorded sufficiently accurate after filtering to allow a feedback control of the feed rate.

**Figure 5.**
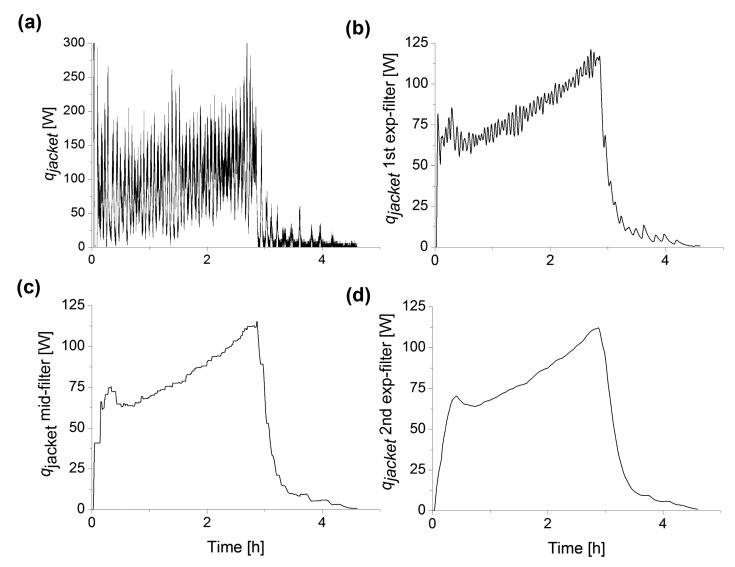
Effect of sequential signal filtering. (**a**) Unfiltered *q_jacket_* signal from model (Equation (3)); (**b**) Filtered *q_jacket_* signal after the first exponential filter with a filtering time of 300 s; (**c**) Filtered *q_jacket_* signal after the middle-value filter with a filter time of 420 s; (**d**) Filtered *q_jacket_* signal after the second exponential filter with a filter time of 300 s.

**Figure 6.**
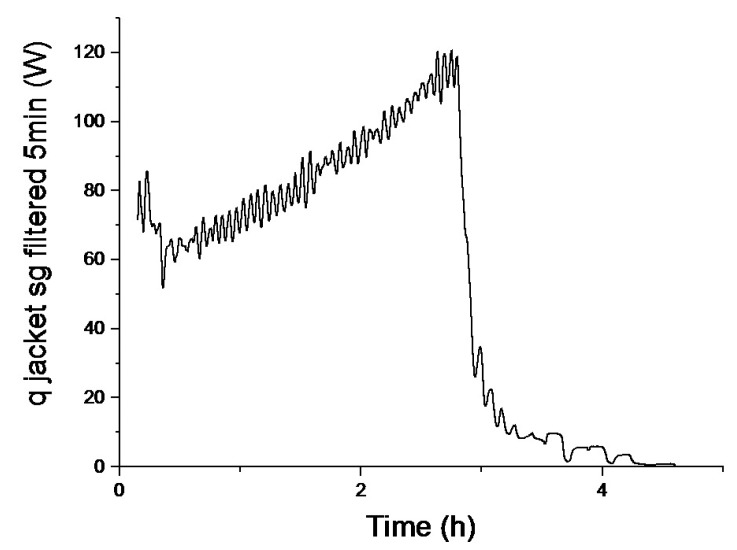
Filtering using a moving-average Savitzky-Golay filter with a filter time of 300 s (the same data set as shown in Figure 5 are used).

Exponential filters *i.e.*, first order low-pass filters with different filter times, in a moving average procedure were therefore applied to the *q_jacket_* signal derived from Equations (1)–(11) (Figure 5). These filters were also compared with a Savitzky-Golay filter with a 5 min data point sampling interval (Figure 6), similar to previous moving average filtering applied to calorimetric monitoring of bioreactors [18]. The filter computes an average value for the center data point by weighing the surrounding set or interval of data points with coefficients computed from solving the least squares equations for the set of data points in the interval.

However, when comparing the Savitzky-Golay to exponential filtering with similar filter times and filter intervals (5 min), the filtering was not better at smoothing oscillations with long period times, (*cf.* Figures 5b and 6). Even with sampling interval above 15 min, e.g., 30 min, the *q_jacket_* signal was distorted, but still not monotonically increasing. Since no single filter was able to make the *q_jacket_* signal monotonically increasing, a sequence of filters was designed and applied to the jacket heat signal (Figure 3). The filters were aligned consecutively, the second filter processed the output of the first filter, and the third filter processed the output of the second filter. Filter times *T_f_* of the filters were easily changed by the user in the soft sensor interface (Figure 4).

The sequential filter was further characterized from its transfer function in simulation. The simulation showed a 97.5% fit with the actual data from the filter when using the transfer function:

(12) $$H(s)=\frac{-0.000104\times s+5.148\times 10^{-6}}{s^{2}+0.004039\times s+5.155\times 10^{-6}}$$

**Figure 7.**
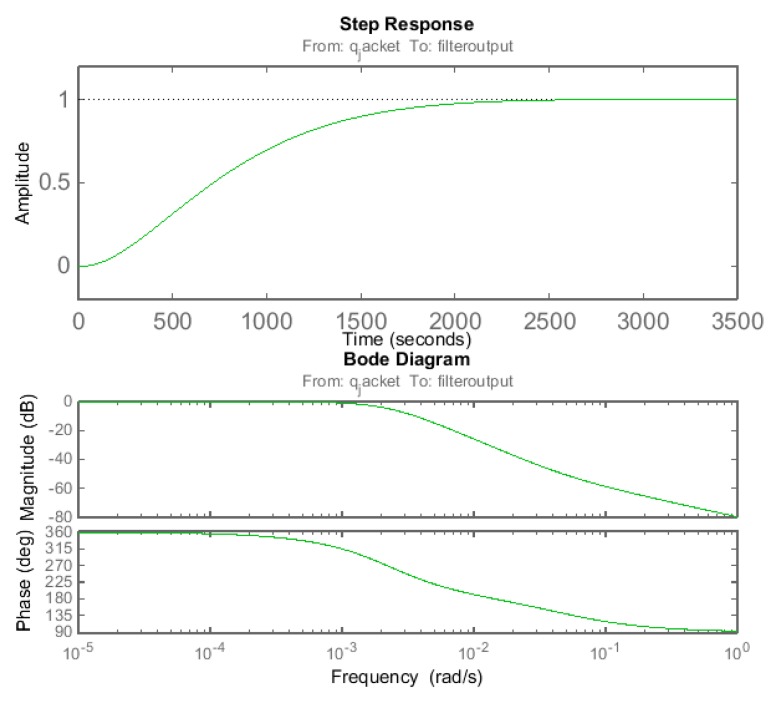
Characterization of the sequential filter based on the model in (Equation (12)). The upper diagram shows a step response of the sequential filter with amplitude of the filter output on the y-axis, expressed as portion of maximum filter output amplitude. The lower diagram shows Bode plots of the filter. Upper panel shows how the magnitude (dB) of the *q_jacket_* signal is attenuated for the different noise frequencies that form the signal. Lower panel shows how the frequencies of the *q_jacket_* signal is phase shifted by the sequential filter.

The model was also analyzed in step responses and Bode plots as shown in Figure 7. As seen in the graphs the sequential filter introduced a modest signal time delay and phase shift in the *q_jacket_*, a condition still making a controlled feeding of the bioreactor culture demanding.

The overall effect of the sequential filter setup is one of a low-pass filter, as signals with noise frequencies below 10^−3^ pass through the filter unchanged, as can be inferred from that the Bode diagram has a straight line at 0 for these frequencies.

As can be seen in Figure 5c, the middle-filter displaces the heat curve to some extent during the feed phase due to the filter delay. This signal is then is further smoothened by the second exponential filter with the same design as the first one (input equation number of the first filter). Figure 5d shows the heat signal after the third sequential filtering.

Thus, the sequential filtering approach we have applied here provides a filter simple to apply and adjust to the existing needs with respect to noise level and culture conditions. The noise reduction observed already after the first stage exponential filter was significant (Figure 5b). After the middle-value filter and the second exponential filter the level of noise in the signal was very low making it favorable for use as input in a PI control of feed rate.

Thus, the combination of three sequential filtering steps showed better performance than other exponential or low pass filter that could smooth the *q_jacket_* signal. In addition, the very easy implementation of our filtering method into the soft sensor structure made it the preferred method.

### 5.2. Monitoring Specific Growth Rate and Biomass Concentration with the Soft Sensor Model

The soft sensor configuration was used to monitoring specific growth and biomass in several fed-batch cultivations with the recombinant *E. coli* carrying the GFP vector.

**Figure 8.**
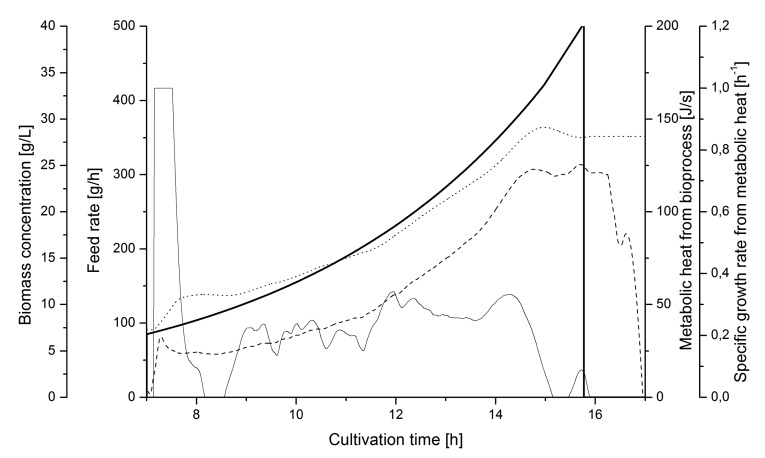
Uncontrolled fed-batch cultivation. Pre-set feed rate ( 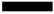), metabolic heat production (⁃⁃⁃), estimated *μ_metabol_* from the soft sensor (—), and biomass from heat (⋯⋯).

Figure 8 shows a representative cultivation chart where the culture is un-controlled during the feed phase. The feeding rate followed a pre-set profile according to a defined exponential function. The *μ_metabol_* changed between 0 and 0.3 h^−1^ during the feed phase. It is evident from the *μ_metabol_* estimates that control is needed if a stable specific growth should be maintained during the whole cultivation.

**Figure 9.**
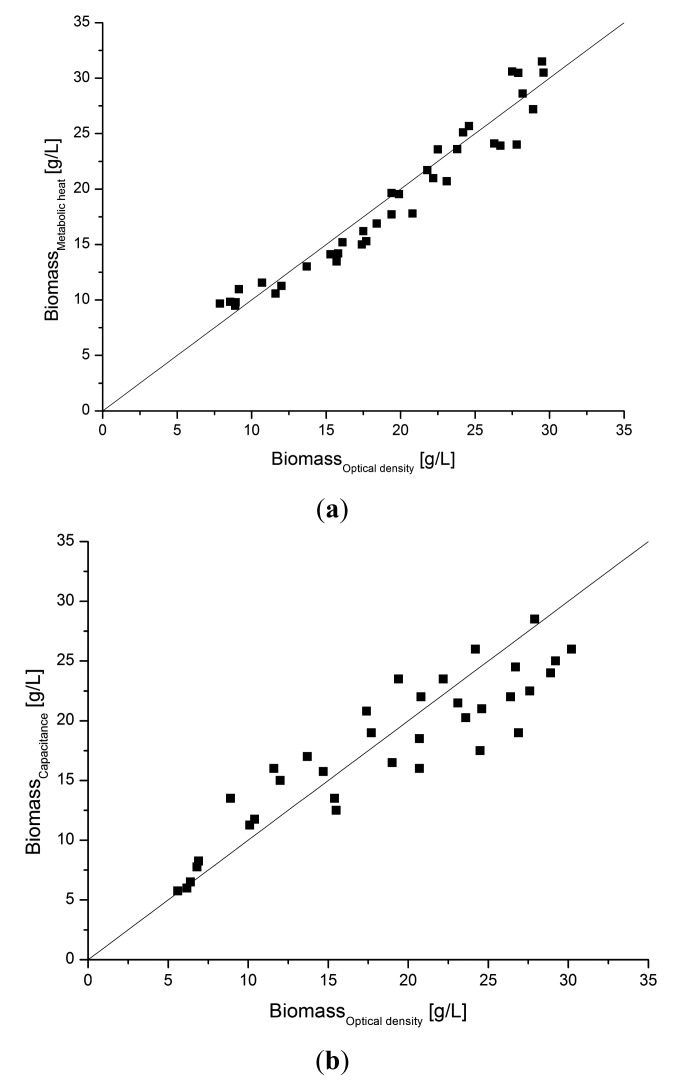
Correlation data collected from four fed-batch cultivations. (**a**) Biomass estimated by the soft sensor model compared with off-line determined biomass; (**b**) Biomass concentration determined by capacitance sensor compared with off-line determined biomass.

In order to compare the signal quality with another soft sensor alternative, a capacitance probe, the estimated heat data from the soft sensor model were collected from several other similar cultivations and compared with off-line samples for optical density converted to dry weight concentration. The correlation between the soft sensor estimates of *X_metabol_* and the *X_optical density_* is shown in Figure 9a. The values coincide satisfactory over the whole range of biomass values during the cultivations. From that correlation we concluded that the metabolic heat soft sensor has good capacity for monitoring as well as control applications.

As an alternative to the metabolic heat soft sensor, an on-line capacitance sensor probe for viable biomass concentration was compared in parallel with the metabolic heat soft sensor. The capacitance probe has previously been applied in yeast and mammalian cell cultures [28–30] as well as in *E. coli* recombinant protein production [31]. Capacitance measurement has also previously been combined with bio-calorimetric monitoring of bioreactors [32]. Thus, the capacitance methodology seems as a realistic alternative to compare our data with.

In Figure 9b the correlation for the capacitance probe is compared with the same biomass off-line data as in Figure 9a. The probe correlates well although the scatter is slightly larger as can be seen. However, the time delay of the capacitance probe signal is much shorter, which is an advantage for control applications.

The capacitance signal originates from the electrical properties of the cell membrane and relates to cell viability [28,29]. Although this is not the same as the metabolic exothermal activity of the culture it indicates that the probe may be a viable alternative to the calorimetric soft sensor setup. However, such a setup requires additional investment in equipment as well as needing more maintenance.

### 5.3. Using the Soft Sensor for Controlling a Fed-Batch Recombinant Protein Production Process

The metabolic heat soft sensor was subsequently used for controlling the feeding of glucose to the bioreactor culture. The bio-calorimetric model [14] has previously been used for adaptive fed-batch control of recombinant protein production in high-density *E. coli* [16] and yeast fed-batch cultivations [17].

**Figure 10.**
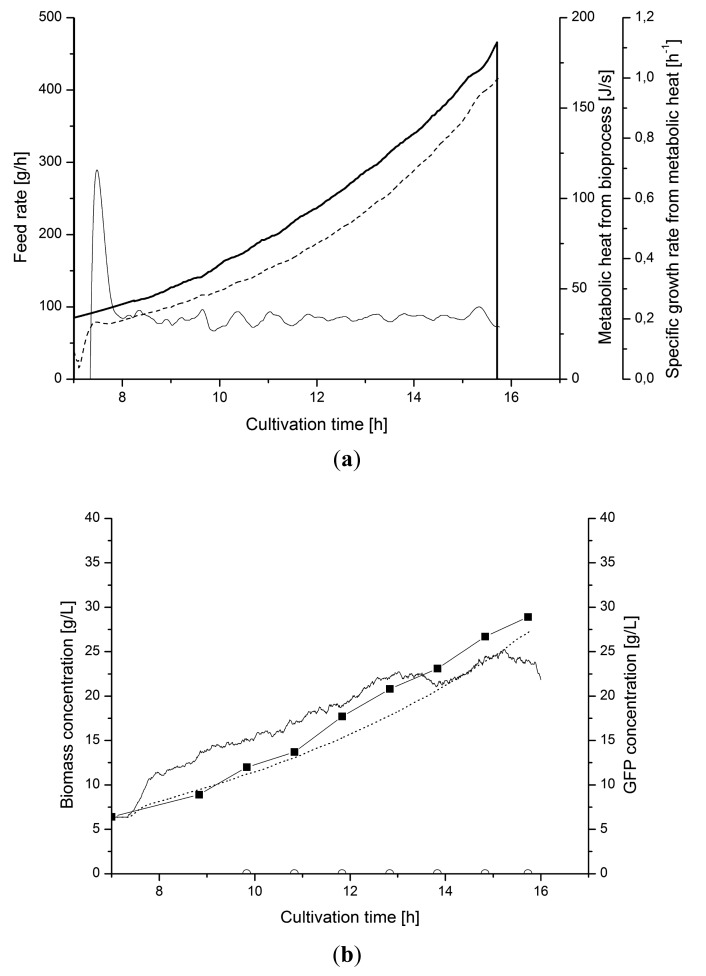
Uninduced fed-batch cultivation. (**a**) Controlled feed rate ( 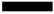), metabolic heat production (⁃⁃⁃) and *μ_metabol_* (—); (**b**) Biomass from heat (·····), optical density (◼) and capacitance (‐‐‐). GFP (○**)** is close to zero.

Here we demonstrated that a low-density cultivation for recombinant protein production with a fivefold lesser production of heat also can be efficiently controlled from on-line metabolic heat calculations. Figure 10 shows an *E. coli* cultivation where the specific biomass growth was controlled by a PI-controller that adjust the feeding of glucose during the fed-batch phase. The parameters of the PI-controller were tuned in separate experiments. The cultivation diagram shows that the controller was able to keep the estimated *μ_metabol_* value constant by minor feed rate adjustments of the exponential feed profile (Figure 10a). The soft sensor also converted the *μ_metabol_* to biomass concentrations *X_metabol_*. This estimate coincided with the measurement by the capacitance probe (*X_cap_*) (Figure 10b).

The applied filtering of the heat signals was necessary for attaining the observed stability. The low heat generation of the culture in the 4–5 L culture volume did not sufficed for attaining a signal stable for enough good control performance. The delay due to the applied filter time made the control more demanding. The tuning of the integration time of the controller was crucial for the stability of the set-point value.

**Figure 11.**
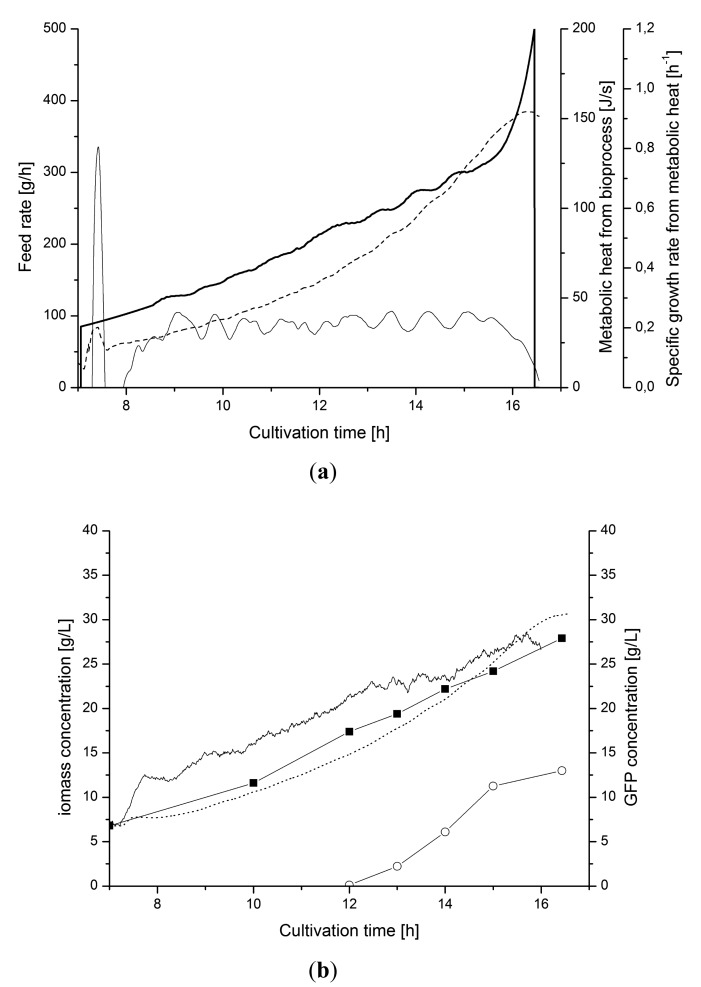
Induced fed-batch cultivation producing recombinant GFP. (**a**) Controlled feed rate ( 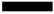), metabolic heat production (⁃⁃⁃) and *μ_metabol_* (—); (**b**) Biomass from heat (·····), optical density (◼) and capacitance measurements (—). The production of GFP (○**)** is induced at 12 h.

Figure 11 shows the control of the cultivation when production of recombinant protein, GFP, was induced. The induction at 12 h disturbed the control due to changing growth behavior. The tuned PI-controller managed to compensate for the disturbance directly and maintained to set-point *μ* value.

This performance was due to the sequential filtering of the signal which provides the required stability for satisfying control with PI-controller.

## 6. Conclusions/Outlook

As previously shown by several researchers, metabolic heat is a useful process variable for on-line estimation of specific growth rate for a variety of organisms growing in a bioreactor. By applying energy balancing from standard temperature sensors in the bioreactor setup the method complies with the PAT concept by combining scientific knowledge and on-line sensing. Many of the previous reports on the use of bio-calorimetry apply the estimation models at favorable conditions, such large bioreactor volumes, where metabolic heat production is significant, and at high cell density, where metabolic heat production is large. However, it is also previously shown that bio-calorimetric monitoring of bioreactors can be applied for heat production at much lower level provided the heat signals are processed by digital filtering methods that reduce noise and improve resolution.

The results presented in this study show that:

- The bio-calorimetric principle can be farther extended to lower biomass concentrations, smaller bioreactors or other technically unfavorable conditions by applying sequential filtering of the estimated signals and, as a result, making the bio-calorimetric approach a more resourceful alternative in small-scale production or to process development.
- On-line capacitance spectrometry shows to be as sensitive for monitoring of growth rate and biomass concentration and therefore can serve as a viable hardware sensor alternative to a bio-calorimetric approach due to its precision and short response time. However, the bio-calorimetric soft sensor approach has the advantage of using existing hardware and does only require the implementation of the bio-calorimetic model with a digital sequential filtering. This favors the bio-calorimetric approach by lesser hardware maintenance and investment.

It may be added that the stability of the model was high once the parameters had been tuned for the controlled path of the cultivation (*i.e.*, the feeding/induction phase as also been noted by others (e.g., [16]). In addition, the key calorimetric parameters were consistent also in all of the phases of the culture. If the yield factors applied in the model would vary an extension of the soft sensor model could be considered by including for example OUR sensor estimates.

Thus, the study demonstrate how the estimation model can be implemented as a soft sensor in a standard computer control software in parallel with other soft sensor functions, and as a result showing a high degree of operability in typical laboratory or small-scale production environments.

The results show that the soft sensor model with the filtering function is comparable, or even better, than alternative on-line hardware sensors for biomass concentration and specific growth rate.

With these results we would like to advocate that the principle of metabolic heat and bio-calorimetry should be considered as a potential PAT tool compatible with the PAT objectives for applying scientific knowledge and on-line sensors in bio-manufacturing.
